# Which digital education approaches most effectively enhance clinical reasoning in undergraduate nursing students: Protocol for a Network Meta-analysis

**DOI:** 10.1371/journal.pone.0347262

**Published:** 2026-04-29

**Authors:** Aeri Jang, Yun-Hee Kim

**Affiliations:** Department of Nursing, Mokpo National University, Yeongsan-ro, Cheonggye-myeon, Muan-gun, Jeollanam-do, Republic of Korea; University of Toronto, CANADA

## Abstract

**Background:**

Clinical reasoning is a core competency in nursing education, enabling students to make accurate and evidence-based clinical decisions. As digital-based learning expands, understanding the comparative effectiveness of different technology-enhanced educational approaches in improving clinical reasoning competence has become essential. However, existing evidence remains fragmented and inconsistent across the intervention types.

**Objective:**

This study aims to systematically evaluate and compare the effects of digital-based education interventions on clinical reasoning competence among undergraduate nursing students using a network meta-analysis.

**Methods:**

This protocol describes a systematic review and network meta-analysis conducted in accordance with the PRISMA-P and Cochrane Handbook guidelines. Randomized controlled trials will be included and analyzed in separate networks. The primary outcome is clinical reasoning competence; secondary outcomes include critical thinking and problem-solving abilities. Searches will be conducted in PubMed, Embase, CINAHL, Web of Science, and CENTRAL from their inception to December 31, 2025. A frequentist network meta-analysis will be performed using R (version 4.1.0) with the netmeta package. Standardized mean differences with 95% confidence intervals will be calculated based on arm-level data. Transitivity and consistency will be assessed using global and local approaches. Intervention rankings will be estimated using P-scores. The certainty of evidence will be assessed using the CINeMA framework and GRADE.

**Conclusions:**

This review will provide a comprehensive synthesis and comparative ranking of digital-based education interventions for improving clinical reasoning and related cognitive outcomes in nursing students. The findings will inform evidence-based decisions regarding the integration of effective digital educational strategies into nursing curricula.

## Introduction

The World Health Organization has identified theory-based clinical reasoning and clinical decision-making abilities as essential core competencies in nursing education, recognizing their strategic role in strengthening professional nursing practice [1]. Clinical reasoning represents a complex cognitive process involving the systematic collection, synthesis, and interpretation of clinical information to support evidence-informed judgments and appropriate interventions, and serves as a fundamental basis for safe and effective nursing care [2]. This underscores the need for educational approaches that can reliably and efficiently foster clinical reasoning during undergraduate nursing education.

As society undergoes rapid digital transformation, educational paradigms must adapt to reflect technology-driven modes of learning. Nursing education, in particular, can no longer rely solely on traditional instructor-centered methods; it must align with this societal shift toward digitalization [3]. In response, a wide range of digital education approaches—including e-learning platforms, web-based learning, mobile applications, and simulation-based digital tools—have been increasingly adopted in undergraduate nursing education. Digital education has demonstrated positive effects on knowledge acquisition, learner engagement, and learning flexibility among nursing and allied health students. It is widely regarded as a pedagogical innovation that supports self-directed learning [4,5].

Recent systematic reviews have demonstrated the effectiveness of diverse teaching strategies—such as simulation-based education, problem-based and case-based learning, debriefing, and mobile-assisted learning—in enhancing nursing students’ clinical reasoning [6]. Likewise, structured training programs have improved students’ clinical judgment and decision-making skills [7]. Despite these positive findings, substantial methodological heterogeneity across studies, including variations in educational designs, assessment tools, and evaluation timing, has hindered the drawing of consistent conclusions. More importantly, most existing reviews have relied primarily on pairwise comparisons, limiting their ability to compare multiple educational approaches simultaneously. Moreover, while digital and technology-enhanced approaches (e.g., virtual simulations, serious games, mobile learning) show promise, their relative effectiveness across different digital modalities remains unclear, as their effects on clinical reasoning are typically reported in isolation rather than in direct or indirect comparison with alternative approaches. Therefore, a systematic review incorporating a network meta-analysis is warranted to compare the relative effectiveness of different digital education approaches and to establish a hierarchy of their effects on clinical reasoning competence among nursing students.

Systematic review and meta-analysis methodologies are essential for synthesizing reliable and objective evidence on educational strategies that enhance nursing students’ clinical reasoning competence in the rapidly expanding field of digital education. Although the psychometric soundness of clinical reasoning assessment instruments has been established [8], standardized frameworks for the comparative evaluation of digital learning interventions remain limited. Simulation-based debriefing and other technology-enhanced instructional approaches have shown potential benefits [9], yet evidence directly comparing the relative effectiveness of digital and conventional educational modalities, as well as among different digital approaches, remains limited. This methodological gap constrains nursing educators’ ability to make evidence-based decisions when selecting and prioritizing digital tools for curricula integration. Given the diversity of digital-based education and the limited direct comparisons among these teaching strategies, traditional meta-analyses are insufficient to evaluate their relative effectiveness comprehensively. Therefore, a network meta-analysis (NMA) is required to integrate direct and indirect evidence, simultaneously compare the teaching strategies, and estimate their relative rankings. To address this issue, the present protocol proposes a systematic review incorporating a network meta-analysis, following the Cochrane Handbook for Systematic Reviews of Interventions [10] and adhering to the PRISMA-P guidelines [11] to ensure transparency, reproducibility, and methodological rigor.

Through a comprehensive synthesis of studies on digital education interventions for enhancing clinical reasoning, this review aims to provide evidence-based insights to support competency-centered nursing education and the effective use of digital learning in academic and clinical settings. Specifically, it seeks to address the following research questions:

What are the relative effects of different digital-based education approaches on clinical reasoning competence among undergraduate nursing students?

Which characteristics of digital-based education interventions are associated with greater effectiveness in improving clinical reasoning?

## Methods

This study adheres to the Preferred Reporting Items for Systematic Review and Meta-analysis Protocols (PRISMA-P) [11], with findings to be reported according to the PRISMA statement [12]. In addition, the reporting of the network meta-analysis will follow the PRISMA extension for network meta-analyses (PRISMA-NMA) [13]. This is a new protocol: no prior version has been registered or published. Any protocol amendments will be documented in PROSPERO (#CRD 420251171748) with a rationale and date.

### Eligibility criteria

This review established eligibility criteria according to the PICOS framework (Population, Intervention, Comparison, Outcomes, and Study design) in alignment with the PRISMA 2020 guidelines [12]. The population of interest comprises undergraduate or graduate nursing students enrolled in accredited nursing programs. Interventions were limited to clearly defined digital-based education approaches explicitly designed to enhance clinical reasoning competence. Eligible digital interventions included distinct digital education modalities, such as learning management system (LMS)–based courses, online or database-supported instruction, digital and virtual simulations, and generative artificial intelligence (AI)–based educational applications.

Studies were included if the intervention outcomes were compared either directly or indirectly with traditional education methods, such as lecture-based instruction, alternative digital education approaches, or no-intervention control groups, allowing the construction of a connected evidence network for network meta-analysis.

The primary outcome of interest was clinical reasoning ability. Secondary outcomes included critical thinking and problem-solving ability, which were considered related but conceptually distinct constructs and were therefore analyzed separately, representing broader cognitive and affective learning outcomes theoretically linked to the development of clinical reasoning.

### Study design

Eligible study designs comprised randomized controlled trials (RCTs) that evaluated the effectiveness of digital education interventions on clinical reasoning. Studies were included if they involved nursing students at the undergraduate level, implemented digital education interventions explicitly targeting the improvement of clinical reasoning, and included at least one comparator arm (e.g., traditional education, no intervention, or alternative digital education approaches), and were published in peer-reviewed journals. No restrictions were placed on the year of publication or language of publication, and non-English studies were eligible provided that sufficient data for effect estimation could be extracted. Studies were excluded if they were dissertations, conference abstracts, grey literature, did not provide adequate data for estimating comparative effects, or involved participants who were not nursing students.

### Information sources

The literature search will follow the Core, Standard, Ideal (COSI) model presented by the US National Library of Medicine [14]. Core databases will include MEDLINE (via PubMed), Embase, and the Cochrane Central Register of Controlled Trials (CENTRAL). Standard databases will include Web of Science, CINAHL, and PsycInfo. An ideal database, Google Scholar, will be used. Search strategies will not be restricted by language, and database indexing terms will be used to capture relevant non-English studies. In addition, the reference lists of all included studies and relevant review articles will be manually screened to identify additional eligible studies.

### Search strategy

Search strategies will incorporate Medical Subject Headings (MeSH) and the Index of Life Sciences Terms (EMTREE), using Boolean operators and truncation to improve retrieval sensitivity and specificity.

Population (Participants) component will use “Students, Nursing [MeSH],” and “undergraduate students [Textword].” For the Intervention component, “Education, Distance [MeSH]”, “Computer simulation [MeSH]”, “Simulation training [MeSH]”, “Virtual reality [MeSH]”, “Avatar [MeSH]”, “Haptic technology [MeSH]”, “Augmented reality [MeSH]”, and “Artificial intelligence [MeSH]” will be used. Text words including “digital education”, “e-learning”, “learning management system (LMS)”, “online learning”, “simulation”, “virtual learning”, “artificial intelligence”, and “generative AI” will be used. The year of publication was not restricted. No restriction on the language of publication will be applied during the literature search. Preliminary search yields from PubMed are presented in Table 1.

**Table 1:** Search Strategy for MEDLINE (PubMed).

No.	Query	Results
1	Students, Nursing [MeSH]	34,428
2	“Nursing Student*”[TIAB] OR “Student, Nursing”[TIAB] OR “Pupil Nurse*”[TIAB] OR “Nurse, Pupil”[TIAB] OR “nurse student*”[TIAB] OR “student nurse*”[TIAB] OR (Nursing[TIAB] AND undergraduate*[TIAB])	32,993
3	#1 OR #2	20,047
4	Education, Distance [MeSH]	7,768
5	“Computer-Assisted Instruction”[MeSH] OR “Online Systems”[MeSH] OR “Internet”[MeSH] OR lms[tiab] OR “learning management system*”[tiab] OR Moodle[tiab] OR Blackboard[tiab] OR Canvas[tiab] OR “e-learning”[tiab] OR “online learning”[tiab] OR “web-based”[tiab]	188,959
6	#4 OR #5	188,656
7	“Online systems” [MeSH] OR “Internet”[MeSH] OR “Databases, Bibliographic”[MeSH]	128,489
8	“online”[tiab] OR “web-based”[tiab] OR “digital education”[tiab] OR “online database”[tiab] OR “online platform”[tiab]	360,984
9	#4 OR #7 OR #8	445,879
10	Computer simulation [MeSH]	340,166
11	Simulation Training [MeSH]	14,012
12	“computer”[All Fields] AND “simulation”[All Fields] OR “computer simulation”[All Fields] OR “simulation”[All Fields] OR “simul”[All Fields] OR “simulate”[All Fields] OR “simulated”[All Fields] OR “simulates”[All Fields] OR “simulating”[All Fields] OR “simulation s”[All Fields] OR “simulation”[All Fields] OR “simulations”[All Fields] OR “simulative”[All Fields] OR “simulator”[All Fields] OR “simulator s”[All Fields] OR “simulators”[All Fields]	1,021,819
13	#10 OR 11 OR #12	1,030,795
14	“Virtual reality”[MeSH] OR “Avatar”[MeSH] OR “Haptic Technology”[MeSH]	9,364
15	virtual realit*[tiab] OR virtual simulation*[tiab] OR educational virtual realit*[tiab] OR instructional virtual realit*[tiab] OR virtual environment*[tiab] OR immersive virtual*[tiab] OR avatar*[tiab] OR haptic*[tiab]	38,739
16	#14 OR #15	39,687
17	“Augmented Reality” [MeSH]	2,049
18	“augmented realit*” [tiab] OR “mixed realit*” [tiab] OR “AR-based” [tiab] OR XR[tiab] OR “extended realit*” [tiab]	12,102
19	#17 OR #18	12,133
20	“Artificial Intelligence”[MeSH]	253,545
21	“Artificial Intelligence”[tiab] OR “Generative Artificial Intelligence”[tiab] OR “Generative AI”[tiab] OR “Artificial Intelligence Chatbot”[tiab] OR “Chatbot*”[tiab] OR “Large Language Model”[tiab] OR “Generative Language Model”[tiab] OR “ChatGPT”[tiab] OR “Perplexity” [tiab] OR “Gemini”[tiab]	97,080
22	#20 OR #21	305,925
23	#6 OR #9 OR #13 OR #16 OR #19 OR #22	1,754,614
24	#3 AND #23	4,432

### Study selection Process

All retrieved records will be imported into EndNote 21 (Clarivate Analytics) for reference management. Duplicate studies will be identified and removed by matching titles, authors, publication years, and Digital Object Identifiers (DOIs), retaining only the most complete version of each article. Non-English studies will be screened and data will be extracted by two reviewers with relevant language proficiency or using translation tools when necessary to minimize language bias.

Two reviewers will independently conduct a two-stage screening process. Titles and abstracts will first be screened against the predefined eligibility criteria, followed by full-text assessment of potentially relevant studies to confirm inclusion. Any discrepancies will be resolved through discussion or consultation with a third reviewer.

**Fig 1:**
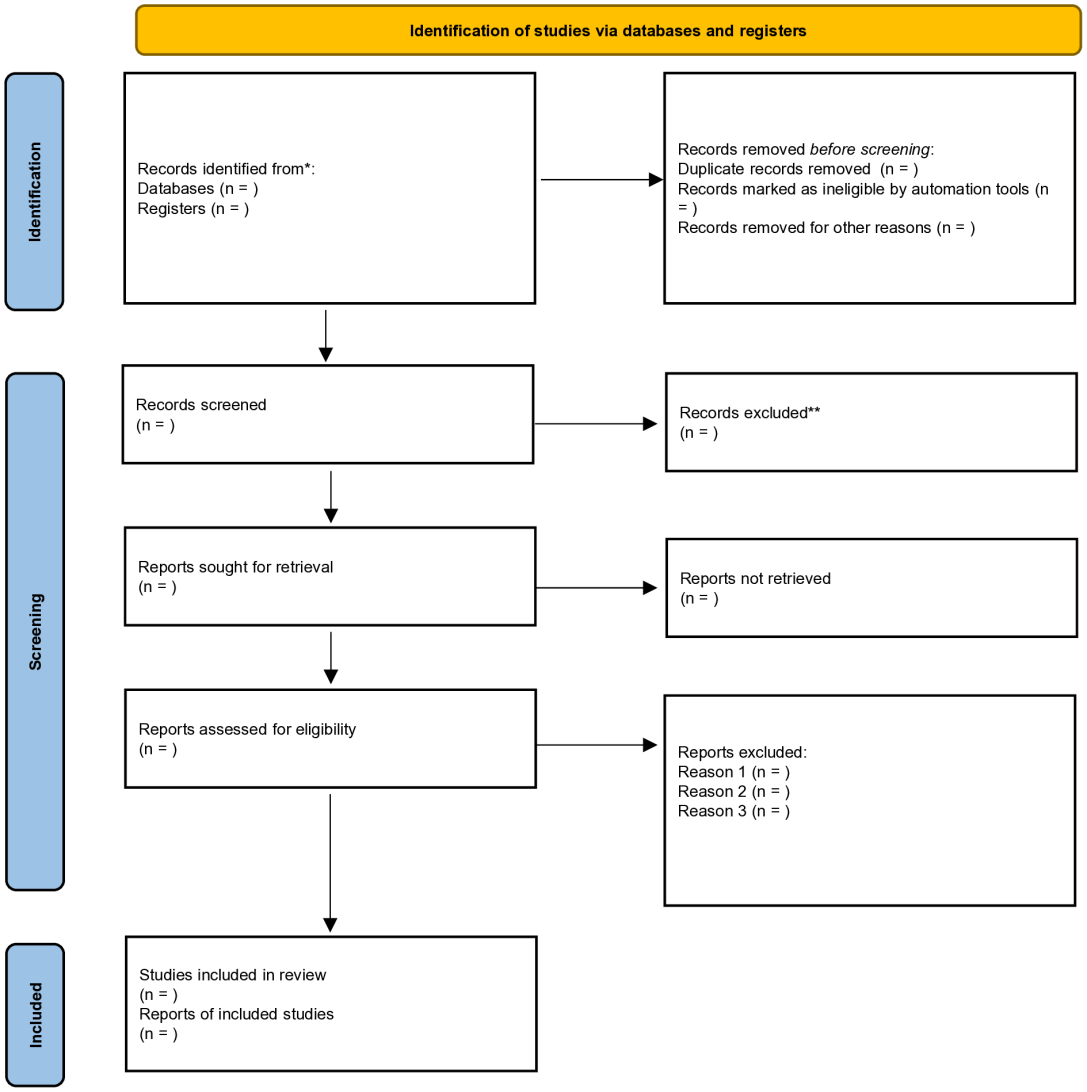
Planned PRISMA 2020 flow diagram for the study selection process.

Data extraction will also be performed independently by two reviewers using a standardized form, which includes study characteristics, participant details, intervention and comparison information, and outcome measures. When study information is incomplete or unclear, the corresponding authors will be contacted to obtain additional details. The overall study selection process will be illustrated using a planned PRISMA 2020 flow diagram (Fig 1). As this manuscript reports a protocol, the number of records at each stage are not yet available and will be reported in the completed systematic review.

### Data extraction

Two reviewers will independently extract data using a predefined and pilot-tested data extraction form. The extracted data will include the following domains:

- Study characteristics: first author, publication year, country, and study design
- Participant characteristics: sample size, academic level (e.g., year in program), and extent of clinical practicum experience
- Intervention details: type of digital-based education (e.g., simulation, virtual reality, serious games, e-learning or LMS, AI-based learning), learning format (individual or group-based; synchronous or asynchronous), duration and frequency of the intervention, feedback mechanism, learning platform or technological tool used, and underlying theoretical or pedagogical framework. Interventions will be categorized into predefined nodes based on their primary technological and pedagogical characteristics to facilitate the NMA. The intervention nodes will include simulation, virtual reality, serious games, e-learning or LMS-based courses, and AI-based learning. Subtypes within the same modality (e.g., high- vs low-fidelity simulation, immersive vs. screen-based virtual reality, or different forms of AI-assisted learning) will be grouped within the same node when they share the same core instructional approaches. When an intervention includes multiple digital components, classification will be based on the dominant educational modality described in the study. Traditional lecture-based education and no-intervention conditions will be considered as reference groups.
- Comparison data: detailed descriptions of comparison conditions, including traditional lecture-based or no intervention, approaches, to enable classification of all interventions and comparators as nodes within the network.

Primary outcomes will include clinical reasoning competence, extracted at the arm level as mean values and standard deviations (SDs) for each intervention and comparator group, along with the measurement instruments used. Secondary outcomes, including critical thinking and problem-solving abilities, will also be extracted to enable secondary network analyses and certainty of evidence assessment. Secondary outcomes will be classified based on the primary construct measured by each assessment instrument. Subscales within multi-domain instruments will not be extracted separately; instead, outcomes will be categorized according to the overall construct defined in the instrument’s original validation study. This approach was chosen to reduce potential misclassification and improve consistency across heterogeneous outcome measures. Variables potentially influencing transitivity will be extracted to assess the comparability of studies across treatment comparisons. Studies lacking sufficient quantitative data for effect estimation, regardless of publication language, will be excluded from quantitative synthesis.

Any discrepancies between reviewers during the data extraction will be resolved through discussion. If consensus cannot be reached, a third independent reviewer or an expert in nursing education will be consulted to make a final decision.

### Outcomes and prioritization

The primary outcome of this review will be clinical reasoning competence, as it represents the core learning goal of digital-based education intervention in nursing. Clinical reasoning constitutes the principal focus of digital-based education interventions aimed at enhancing higher-order cognitive skills in undergraduate nursing students.

Secondary outcomes include critical thinking and problem-solving abilities, which reflect broader cognitive learning effects that help develop clinical reasoning competence. Critical thinking and problem-solving are essential cognitive skills that support clinical reasoning and promote reflective, analytical, and logical decision-making in nursing students.

Clinical reasoning competence has been prioritized as the primary outcome because it reflects the most proximal and practice-relevant indicator of higher-order cognitive learning in nursing education. In contrast, critical thinking, problem-solving, and affective learning outcomes provide complementary evidence of the broader educational impact of digital-based learning interventions.

### Risk of bias in individual studies

The Risk of bias in RCTs will be evaluated using the Cochrane Risk of Bias 2.0 (RoB 2.0) tool, developed by the Cochrane Bias Method Group, which assesses five domains: (1) randomization process, (2) deviations from intended interventions, (3) missing outcome data, (4) measurement of the outcome, and (5) selective reporting. Each literature will be assessed using domain-specific signal questions and algorithm-based judgments, with the risk of bias categorized as “low risk,” “some concerns,” or “high risk.” Based on responses to the signal questions within each domain, the degree of bias will be rated as “low,” “some concerns,” or “high” according to the evaluation algorithm outlined in the RoB 2.0 guidance. Overall bias assessments will be determined as “low,” “some concerns,” or “high” using the criteria specified in the RoB 2.0 framework [15].

The overall risk of bias is determined by the highest level of bias identified across domains. Two researchers will independently assess all studies using a pre-piloted form, with inter-rater agreement calculated and discrepancies resolved through consensus or arbitration by a third reviewer. Findings from the bias assessments will inform the interpretation of the pooled estimates and will be explored in the sensitivity analysis by excluding studies judged to be at high or critical risk of bias. A summary of finding table will be constructed to present the estimated effect sizes (standard mean differences, SMD) and 95% confidence intervals (CI) for each digital-based education intervention compared with lecture-based education or no-intervention controls. The certainty of evidence will be assessed using the CINeMA framework in conjunction with GRADE. The planned structure of the summary of finding table is presented in Table 2.

**Table 2:** Planned Summary of Findings Table of Network Meta-analysis.

Intervention	Comparator	Outcomes	Effect size (SMD)	95% CI	Certainty of evidence (CINeMA/ GRADE)
Simulation	Lecture-based education	Clinical reasoning competence			
		Critical thinking			
		Problem-solving ability			
	No intervention	Clinical reasoning competence			
		Critical thinking			
		Problem-solving ability			
Virtual reality	Lecture-based education	Clinical reasoning competence			
		Critical thinking			
		Problem-solving ability			
	No intervention	Clinical reasoning competence			
		Critical thinking			
		Problem-solving ability			
Serious games	Lecture-based education	Clinical reasoning competence			
		Critical thinking			
		Problem-solving ability			
	No intervention	Clinical reasoning competence			
		Critical thinking			
		Problem-solving ability			
E-learning /LMS-based course	Lecture-based education	Clinical reasoning competence			
		Critical thinking			
		Problem-solving ability			
	No intervention	Clinical reasoning competence			
		Critical thinking			
		Problem-solving ability			
AI-based learning	Lecture-based education	Clinical reasoning competence			
		Critical thinking			
		Problem-solving ability			
	No intervention	Clinical reasoning competence			
		Critical thinking			
		Problem-solving ability			

### Data synthesis

The meta-analysis will be conducted following the Cochrane Handbook for Systematic Reviews of Interventions (version 6.4) [10]. For the primary outcome and secondary outcomes, the mean, SDs, and sample size will be extracted at the arm level for each intervention and comparator group. When SDs are not directly reported, they will be calculated from standard errors, CI, p-values, or t-statistics where possible. If outcome data are reported only graphically, numerical values will be extracted using validated digital extraction tools. Studies for which dispersion measures cannot be obtained or reliably estimated will be narratively synthesized or excluded form quantitative analyses. Network effect will be estimated separately for RCTs and NRCTs, presented as SMDs with 95% CIs. All outcome measures will be oriented in the same direction prior to analysis, such that higher SMD values consistently indicate greater improvement in clinical reasoning competence. When necessary, outcome scales will be reverse-coded to ensure interpretability and comparability across studies.

All statistical analyses will be performed using R software (version 4.1.0; R Foundation for Statistical Computing, Vienna, Austria). A frequentist network meta-analysis will be conducted using the ‘netmeta’ package. If the network structure does not meet the minimum connectivity requirements for NMA, pairwise meta-analyses or narrative synthesis will be conducted as appropriate. Minimum connectivity will be defined as a network in which all interventions are connected directly or indirectly through at least one common comparator, allowing estimation of relative treatment effects across the network.

Intervention rankings will be estimated within each network using P-scores. Rankings across networks will be interpreted cautiously, focusing on the concordance of relative effects rather than direct numerical comparisons. P-scores will be interpreted as probabilistic summaries of relative treatment performance and will not be considered substitutes for effect size magnitude or clinical relevance.

Transitivity will be assessed by examining the distribution of prespecified potential effect modifiers across treatment comparisons (e.g., age, baseline clinical reasoning competence level, year of study or clinical exposure, intervention intensity and duration, learning format, and outcome measurement instruments). In addition, the year of study or level of clinical exposure will be considered as a potential effect modifier in exploratory analyses. Where sufficient data are available, network meta-regression analyses will be conducted to examine whether differences in academic level or clinical exposure influence the effectiveness of digital-based education interventions. When studies include mixed student populations across multiple academic years, classification will be based on the predominant academic level reported in the study, or otherwise categorized as mixed undergraduate populations. Consistency between direct and indirect evidence will be evaluated within each network using global and local approaches (e.g., design-by-treatment interaction and node-splitting/loop-specific methods, as applicable). The design-by-treatment interaction model will be used as the primary global test to assess overall inconsistency in the network. Local inconsistency will be further explored using node-splitting or loop-specific approaches to identify specific comparisons contributing to inconsistency. If significant inconsistency is detected, potential sources will be explored through sensitivity analyses, subgroup analyses, or network meta-regression based on prespecified effect modifiers.

Statistical heterogeneity will be assessed using the chi-square (χ²) test, Higgins’ I² statistic, and between-study variance (τ²). In addition, 95% prediction intervals will be reported to enhance the extrapolability of findings. Heterogeneity will be interpreted as low (I² ≤ 25%), moderate (25–75%), or high (>75%). In addition, inconsistency between direct and indirect evidence will be evaluated at the network level. Given the expected diversity of interventions, a random-effects model will be used with the inverse-variance method. Multi-arm trials will be handled using methods that account for the correlation between effect estimates within studies, as implemented in the netmeta package. Specifically, the variance-covariance structure of treatment effects will be appropriately modeled to avoid double-counting of shared comparators. When outcome data are available at multiple time points, post-intervention effects will be prioritized for the primary analysis, and follow-up effects will be analyzed separately where data permit. When adjusted estimates (e.g., ANCOVA-adjusted effects) or change scores are reported, these will be prioritized over unadjusted post-intervention values. Sensitivity analyses will be conducted to evaluate the influence of individual studies, including leave-one-out analyses and the assessment of potential outliers. Where information is available, additional sensitivity analyses will be performed excluding studies that used non-validated instruments for assessing clinical reasoning competence.

To explore potential sources of heterogeneity and to address the secondary research question regarding intervention characteristics associated with greater effectiveness, prespecified subgroup analyses and network meta-regression analyses will be conducted based on study-level characteristics. Covariates included in meta regression analyses will be selected a priori based on theoretical relevance, empirical support from previous studies, and data availability across the network. Meta-regression analyses will be conducted when a sufficient number of studies are available (typically k ≥ 10–15). Sensitivity analyses will be performed by excluding studies judged to have a high risk of bias.

Where sufficient data are available, small-study effects and potential publication bias will be explored using comparison-adjusted funnel plots. The certainty of evidence across the network will be assessed using an established framework for NMA, such as Confidence in Network Meta-Analysis (CINeMA) [16] and GRADE.

Variables extracted as potential effect modifiers will be used to assess the transitivity assumption and, where appropriate, further explored through NMA.

### Metabias

Publication bias will be evaluated through visual and statistical methods to ensure the validity of the synthesized findings. For the comparison-adjusted funnel plot, treatment arms will be ordered prospectively, with traditional lecture-based education or no-intervention defined as the reference comparator, while alternative digital-based education approaches will be ordered relative to this reference within the network structure. Where appropriate and when a sufficient number of studies are available, Egger’s regression test [17] will be applied to quantitatively assess funnel plot asymmetry by examining the significance of the regression intercept. Selective outcome reporting bias will be assessed by comparing reported outcomes with registered study protocols or trial registration records, when available. Potential language bias and small-study effects will also be explored where feasible. These assessments will inform both the interpretation of the synthesized results and the evaluation of certainty of evidence at the network level.

### Confidence in Cumulative Evidence

The certainty of evidence for all network estimates will be assessed using the CINeMA framework [17], which operationalizes the principles of the Grading of Recommendations Assessment, Development and Evaluation (GRADE) [10] and is specifically designed for evaluating confidence in the results of network meta-analysis. Certainty assessments will be conducted separately for each outcome, including the primary outcome (clinical reasoning competence) and the secondary outcomes (critical thinking and problem-solving abilities), as well as categorized as high, moderate, low, or very low. In addition, the GRADE approach will be considered alongside CINeMA to provide complementary evaluation of the certainty of evidence and to enhance the transparency of the quality assessment process.

CINeMA evaluates confidence in network estimates across six domains: within-study bias, reporting bias, indirectness, imprecision, heterogeneity, and incoherence. Because this review includes only RCTs, the certainty of evidence will initially be considered at a high level and may be downgraded based on the CINeMA evaluation domains.

Within-study bias will be assessed based on the methodological quality of the studies contributing to each network comparison and their relative contributions to the network estimates. Reporting bias, corresponding conceptually to the across-studies bias domain in the GRADE framework, will be evaluated by considering evidence of selective publication, selective outcome reporting, and small-study effects, informed by comparison-adjusted funnel plots and related statistical assessments where applicable. Indirectness will be assessed by evaluating the applicability of the study populations (undergraduate nursing students), digital-based education interventions, and outcome measures to the review question, with particular attention to the transitivity assumption underlying the NMA. Imprecision will be assessed based on the width of 95% CIs around network effect estimates, whether they include a no-effect estimate, and the magnitude and direction of estimated effects on educational outcomes.

Given the diversity of outcome measurement instruments and the absence of established minimal essential differences for cognitive outcomes such as clinical reasoning, critical thinking, and problem-solving abilities, judgements of imprecision will focus on statistical uncertainty and interpretability of effect sizes rather than predefined thresholds. Heterogeneity will be evaluated using estimates of between-study variance (τ²) and prediction intervals. Incoherence will be assessed by comparing direct and indirect evidence within the same network and by examining inconsistencies across different sources of evidence within the network.

A summary of findings table will be presented to report network effect estimates, 95% CIs, and the certainty of evidence for each outcome. Two reviewers will independently conduct the CINeMA and GRADE assessments, and any disagreements will be resolved through discussion or consultation with a third reviewer.
